# Round the Hospitals

**Published:** 1921-05-21

**Authors:** 


### ROUND THE HOSPITALS.

The case of the nurses suspended from duty at the Steyning Infirmary presents one of those perplexing situations which from time to time occur in institutions. The two probationers had practically completed their course, and had passed their examination. At this crucial moment they were both guilty of acts of indiscipline, "gross unkindness to one of the patients," and insubordination, whereupon they were summarily dismissed by the medical officer. The superintendent nurse made no charge against them beyond "little trifles," "a little friction." The matter has been thoroughly investigated and the charges have been substantiated. Should they or should they not be deprived of their certificate? The medical officer declines to sign it, but a compromise has been agreed on. If the examiner will sign, the chairman will witness it. We incline to think this is the better course.

News is to hand of the retirement of the Matrons of Portsmouth's two largest hospitals, Miss Alcock from the Royal and Miss Foyster from the Union Infirmary. Both have served their institutions long and devotedly and their retirement cannot be grudged to them after the strenuous war years. In Miss Alcock's case as Principal Matron of the 5th Southern Territorial Hospital a considerable amount of organising work fell to her share, in recognition of which she was awarded the Royal Red Cross. Before coming to Portsmouth in 1910 she was assistant matron at Derbyshire Royal Infirmary. Miss Foyster, who has been matron of Portsmouth Union Infirmary since 1906, was trained at Whitechapel Infirmary, proceeding from there to the Poplar and Stepney Sick Asylum, now known as St. Andrew's Hospital, Bow.

At St. Mary's Hospital, Paddington, arrangements are being made to accommodate twenty nurses in premises in Norfolk Square, where some of the rooms are being divided into cubicles. The new nurses' quarters have long been urgently required, and Belleville Ward, which has been used for a nurses' dormitory, will now be set free for addition to the new midwifery department. Fifteen nurses, out of eighteen certificated by the hospital during the year, passed the C.M.B. examination, and three have been promoted to ward sisters. On the resignation of Miss Morrison, who was so well known and highly esteemed in the office of home sister, which she held for many years, the post of home sister was combined with that of sister-tutor, an arrangement which should prove highly satisfactory provided the requisite practical talents can be found associated with teaching ability. In a moderate-sized institution the duties of the one post supplement those of the other, so that possible friction and waste of time are avoided.

Dr. A. K. Newman has created something of a sensation by asserting in the New Zealand House of Assembly during a debate on the Hospitals Amendment Bill that it was distinctly proved during the war that the New Zealand nurses were better qualified and had a wider knowledge of nursing work than any other nursing section in the field. This is the right spirit, and we are sure that Canadian, Australian, English, Scotch, Welsh, and Irish doctors, to say nothing of American, would be prepared to enter the field on the same footing and back their own nurses against the world. What we like about Dr. Newman is his conviction that the standard can only be maintained by making conditions more attractive, by giving the nurses less work, more holidays, and better pay.

The Metropolitan Asylums Board has decided after full inquiry into all the aspects of the case to permit an extension of the living-out system to all members of the mental hospitals staffs. In institutions containing accommodation actually provided for the use of the staff this must be utilised in one way or another, but as nearly all the mental hospitals are suffering from overcrowding this will not prove a great bar to the living-out scheme. Up till now non-residents have been boarded in the institutions on payment of 2s. 4d. a day, but it is now proposed they shall make their own messing arrangements, suitable rooms being provided. With an efficient mess committee the nurses ought to be able to provide for themselves such meals as fall within their duty hours to their complete satisfaction.

The matron's salary at the M.A.B. Southern Hospital has been raised to a higher grade, and now stands at £249, rising annually by £10 to £299 per annum. The normal accommodation is 1,532 beds in all, divided between the Upper and Lower Hospitals, which are some distance apart. The female staff numbers 617. It is the matron's duty to see to the equipment as regards blankets, sheets, staff uniforms, patients' clothing, etc., and it is placed on record that the present matron showed conspicuous ability and organising powers of a high order in re-equipping the Lower Hospital for the reception of infectious patients in the record epidemic last year. Emoluments of board, lodging, and washing and the bonus of £18 per annum remain as before.

At the annual prize-giving at the Islington Infirmary Sir George H. Makins, G.C.M.G., C.B., had a word of warm commendation for the old nurses before the period of high training. "Many were most observant, most kind, and most efficient." He strongly objected to the nurses of fifty years ago being classed as Sairey Gamps. It is quite clear, however, that Sir George fully appreciates the value of a sound nursing education such as is afforded to probationers at the Highgate Hill Infirmary.

It is pleasant to learn that Viscountess Cowdray has received many letters from nurses regarding her gift to the College of Nursing, which we may remark incidentally cannot fail to benefit the whole nursing profession. She desires through these columns to acknowledge the very large number of kind and appreciative letters received from individual nurses and nursing organisations at home and abroad regarding the gift of 20 Cavendish Square as a club for nurses and headquarters of the College of Nursing. It is impossible to reply to each letter individually, but Viscountess Cowdray wishes to record the pleasure which it has given her to know, from members of the profession, that such a club and headquarters is so welcomed and valued.

The quiet days of prayer and study and the pleasant social gatherings of the Nurses' Missionary League at University Hall, Gordon Square, do much to keep alive a keen spirit of interest in its members. The annual Conference held on Wednesday, May 11, was a particularly successful and helpful one: the members must have found much that was encouraging to them in the Demonstration Study Circle given by nurses from Guy's, the London, St. Thomas's, and the Prince of Wales's hospitals. Mr. Henry White, M.R.C.S., spoke of his work in Persia and the need for more nurses for the hospitals. Photographs of many happy holidays spent at Sandsend in camp, of bathing parties and picnics, were in evidence, and in the afternoon songs and recitations formed a large part of the programme. To all the day must have brought a greater knowledge of the activities of the League; to some it may have meant even more: it may have awakened the first stirrings of desire to carry to foreign lands lessons of health and healing both spiritual and physical.

The Fever Nurses' Association has done good service in bringing the question of salaries before the District Hospitals. At the recent meeting of the Swindon and District Hospital Board, the following recommendations, arising out of the circular letter received from the Association, were adopted: "That after full three years' probationary period a nurse remaining in the service of the Board shall receive £50, rising to £60 per annum; that the salary of Sister McLennon be increased from £70 to £90 per annum, rising by annual increments of £10 to £120 per annum; and that the salary of the matron be increased from £150 to £200 per annum, rising to £250." Some discussion arose on the question of the matron's salary, which a few years ago was £50 to £60. But it was evident from the remarks of the medical officer and other members of the Board that the services of the matron are highly appreciated, and the rise in salary was passed with great cordiality.

